# Associations of Genetically Predicted Vitamin B_12_ Status across the Phenome

**DOI:** 10.3390/nu14235031

**Published:** 2022-11-26

**Authors:** Marie-Joe Dib, Kourosh R. Ahmadi, Loukas Zagkos, Dipender Gill, Brooke Morris, Paul Elliott, Abbas Dehghan, Ioanna Tzoulaki

**Affiliations:** 1 Department of Epidemiology and Biostatistics, School of Public Health, Imperial College London, London SW7 2BX, UK; 2 British Heart Foundation Centre of Excellence, Imperial College London, London SW7 2BX, UK; 3 Department of Nutritional Sciences, School of Biosciences and Medicine, University of Surrey, Guildford GU2 7XH, UK; 4 Baylor Biology Department, Baylor University, Waco, TX 76706, USA; 5 Dementia Research Centre, Imperial College London, London SW7 2BX, UK; 6 Department of Hygiene and Epidemiology, University of Ioannina School of Medicine, Ioannina 45110, Greece

**Keywords:** vitamin B_12_, deficiency, epidemiology, Mendelian randomisation, pernicious anaemia

## Abstract

Variation in vitamin B_12_ levels has been associated with a range of diseases across the life-course, the causal nature of which remains elusive. We aimed to interrogate genetically predicted vitamin B_12_ status in relation to a plethora of clinical outcomes available in the UK Biobank. Genome-wide association study (GWAS) summary data obtained from a Danish and Icelandic cohort of 45,576 individuals were used to identify 8 genetic variants associated with vitamin B_12_ levels, serving as genetic instruments for vitamin B_12_ status in subsequent analyses. We conducted a Mendelian randomisation (MR)-phenome-wide association study (PheWAS) of vitamin B_12_ status with 945 distinct phenotypes in 439,738 individuals from the UK Biobank using these 8 genetic instruments to proxy alterations in vitamin B_12_ status. We used external GWAS summary statistics for replication of significant findings. Correction for multiple testing was taken into consideration using a 5% false discovery rate (FDR) threshold. MR analysis identified an association between higher genetically predicted vitamin B_12_ status and lower risk of vitamin B deficiency (including all B vitamin deficiencies), serving as a positive control outcome. We further identified associations between higher genetically predicted vitamin B_12_ status and a reduced risk of megaloblastic anaemia (OR = 0.35, 95% CI: 0.20–0.50) and pernicious anaemia (0.29, 0.19–0.45), which was supported in replication analyses. Our study highlights that higher genetically predicted vitamin B_12_ status is potentially protective of risk of vitamin B_12_ deficiency associated with pernicious anaemia diagnosis, and reduces risk of megaloblastic anaemia. The potential use of genetically predicted vitamin B_12_ status in disease diagnosis, progression and management remains to be investigated.

## 1. Introduction

Vitamin B_12_, also known as cobalamin, is an essential micronutrient that plays a pivotal role in human health by acting as a cofactor for two enzymes [1]. In the cytoplasm, vitamin B_12_ facilitates the re-methylation of homocysteine to methionine via methionine synthase. In the mitochondria, vitamin B_12_ acts as a cofactor in the conversion of methyl malonyl-CoA to succinyl-CoA by the enzyme methyl malonyl-CoA mutase, which is essential for energy production. There is significant variability in vitamin B_12_ plasma levels explained by environmental (dietary intake, atrophic gastritis affecting absorption) and heritable factors. In fact, 56% of the variability in plasma vitamin B_12_ levels is attributable to genetic factors (*h*^2^ = 56%) [2]. 

Clinical vitamin B_12_ deficiency, manifested as megaloblastic anaemia, is associated with a range of health outcomes, such as impaired cognitive decline and cardiovascular disease [3], while the health consequences of sub-clinical deficiency remain unknown [3,4]. Vitamin B_12_ deficiency, defined as circulating B_12_ levels below 148 pmol/L [5], occurs in 6% of individuals aged less than 60 and 20% in those aged more than 60, and its prevalence is increasing, especially in older adults [6,7,8]. As the population ages, there is a growing need to redress vitamin B_12_ deficiency and its important clinical sequelae. Observational studies on the role of vitamin B_12_ status on disease risk can be hindered by confounding from unmeasured and unknown environmental factors and reverse causation bias from outcomes that affect vitamin B_12_ status. The use of vitamin B_12_ status-related genetic variants can overcome these limitations because their random allocation during conception minimizes confounding, and their presence from birth prevents reverse causation. Mendelian randomisation (MR) can also be applied in an agnostic exploration of traits across the human phenome, often termed as MR–phenome-wide association study (MR–PheWAS) [9]. This method has been suggested as a hypothesis-generating approach which aims to explore potential causal relationships between an exposure of interest and a wide range of diseases, and aids in the generation of novel hypotheses on the shared genetic aetiology of related phenotypes.

With its wealth of genotypic and phenotypic data collected in large numbers, the UK Biobank provides a unique opportunity to agnostically scrutinise potential health outcomes from a range of 945 diseases that are attributable to vitamin B_12_ status. To this end, we conducted a MR-PheWAS of vitamin B_12_ status using data from the UK Biobank, with the aim of understanding the clinical consequences of variation in lifelong vitamin B_12_ status through genetic predisposition. 

## 2. Materials and Methods

### 2.1. Study Design

This study implemented two-sample MR-PheWAS analyses to identify causal associations between genetically predicted vitamin B_12_ status and clinical health outcomes. 

### 2.2. Study Populations

The UK Biobank is a large-scale, population-based, prospective cohort with more than 500,000 participants aged 40–69 years are enrolled [10]. The study has collected biological samples and wide range of phenotypic data from its participants, including data from questionnaires, physical measures, sample assays, genome-wide genotyping and longitudinal follow-up for a plethora of health-related outcomes. National health records have been linked with the baseline and genotypic data. Genotypic and phenotypic data used in this study were obtained from the UK Biobank under an approved data request application (application ID: 236). The processes for genotyping and data management have recently been described in depth [11]. UK Biobank has ethics approval from the North West Multi-Centre Research Ethics Committee (11/NW/0382).

### 2.3. Selection of Genetic Instruments Characterising Vitamin B_12_ Status

Our exposure of interest was vitamin B_12_ status, measured clinically using serum vitamin B_12_ measurements as a biomarker. We selected the SNPs for serum vitamin B_12_ based on an Icelandic and Danish sequencing initiative that has reported 11 loci associated with serum vitamin B_12_ levels at genome-wide significant level (*p* < 5 × 10^−8^) [12]. This is the largest genome-wide association study of vitamin B_12_ status to date. Of them, two SNPs (rs602662 (*FUT2*) and rs778805 (*FUT6*)) are known to have pleiotropic effects, as FUT2 is known to determine ABH antigen secretor/non-secretor status and FUT6 is involved in the creation of Lewis antigens. They were excluded from the MR-PheWAS to abide by MR assumptions. One SNP, rs12272669, was not available in our dataset and was excluded. Using PLINK [13], we computed a weighted genetic risk score (GRS) of vitamin B_12_ status using the 8 genetic instruments (rs2270655, rs1141321, rs1801222, rs34324219, rs41281112, rs3742801, rs2336573, rs1131603) described in Table 1 to perform the PheWAS analyses. The approach used by PLINK has been previously described [14]. Used genetic data had been previously quality controlled as described in detail in https://biobank.ctsu.ox.ac.uk/crystal/crystal/docs/genotyping_qc.pdf (accessed on 25 November 2022). After quality control, genetic variants passing a test of Hardy–Weinberg equilibrium (HWE) (*p* > 10^−6^) were considered. Non-European participants were excluded, in addition to individuals with discordant reported sex and genetic sex. The genotypes were coded according to number of serum vitamin B_12_ increasing alleles. Summary and F-statistics to assess the strength of the genetic instruments are shown in Table 1, as previously calculated [12,13,14,15]. 

### 2.4. Statistical Analysis

#### 2.4.1. Phenome-Wide Association Analysis 

The analysis was restricted to 426,295 UK Biobank participants after excluding non-European samples, relatives of first and second degree and samples with sex mismatch. We pooled the available hospital episode data, cancer registry data, and death registry data together and included both the primary and secondary International Classification of Diseases (ICD) codes. The phecode grouping system was used, which includes 1866 hierarchical phenotype codes that could be directly matched to the ICD-9/10 codes through the ‘PheWAS’ R package [16,17]. The scheme automatically excludes patients that have similar or potentially overlapping disease states from the corresponding control group. After mapping diagnostic ICD-9 and ICD-10 codes to phecodes, 945 remained for analysis after filtering out disease outcomes with low prevalence (number of cases < 200) [18]. A series of case–control groups were then generated for each phecode. We conducted a PheWAS of the vitamin B_12_ GRS and separately for *rs601338* in 439,738 individuals from the UK Biobank using logistic regression after adjustment for age, sex and the first 10 genetic principal components. To account for multiple testing, we estimated the false discovery rate (FDR) adjusted *p* values (*p*-values). A q value not greater than 5% was considered significant [19]. 

#### 2.4.2. Mendelian Randomisation Analyses

Two-sample MR analysis was performed for the estimation of the effect of vitamin B_12_ status on the health outcomes identified as statistically significant 5% false discovery rate (FDR) in PheWAS analysis. MR uses genetic exposures as instruments to determine the causal association between an exposure and an outcome of interest. Estimates obtained from MR analyses reflect an unbiased causal estimate if the following assumptions are met: (i) the genetic instruments are associated with the exposure (ii) the genetic instruments are independent of confounders of the exposure-outcome association (iii) the genetic instruments are independent of the outcome. MR estimates for each SNP were derived as the ratio of this (SNP-outcome association) with the corresponding association effect size of the same SNP with serum vitamin B_12_ levels (SNP-exposure association) from previous GWAS on vitamin B_12_ status in a Danish and Icelandic population of 45,576 individuals [12]. Inverse-variance weighted (IVW) meta-analysis of MR estimates for the 8 genetic instruments was conducted to derive the MR estimate for the effect of vitamin B_12_ status on risk of each outcome [20]. Statistical significance of MR effect estimates across all phenotypes was ascertained using the FDR method with a 5% threshold to correct for multiple testing of correlated phenotypes [19]. 

#### 2.4.3. Sensitivity Analyses

We conducted a series of sensitivity analyses to support the validity and to ensure robust causal inference of our MR analyses [21]. Pleiotropy refers to the phenomenon in which genetic instruments affect the outcome of interest through pathways that are partly independent of the exposure, and is a source of potential bias. We applied the MR-Egger method to explore the assumption of no pleiotropy in the selected genetic instruments [22]. The MR-Egger method assumes that the ‘Instrument Strength is Independent of the Direct Effect’, known as the InSIDE assumption, and assesses whether genetic variants have pleiotropic effects on the outcome that differ from zero. It corrects for pleiotropy by introducing a nuisance parameter which quantifies directional pleiotropy, and provides a consistent estimate of the causal effect under a weaker assumption [23]. While this reduces the power of MR-Egger to detect a causal effect, it was considered supportive when the effect estimate was in the same direction as MR-IVW and the MR-Egger intercept was statistically non-significant (*p* > 0.05). 

In addition, we conducted the MR-weighted median method, which returns an accurate causal estimate, provided that least 50% of the weight in the analysis comes from valid instrumental variables [24]. Lastly, heterogeneity in the MR estimates generated by different instrument SNPs beyond that expected by chance can be used to indicate the presence of pleiotropy. We assessed for this in our MR–PheWAS analysis using the Cochran Q test (interpreting *p* < 0.05 as evidence of heterogeneity and thus pleiotropy). We considered associations to be statistically significant if *p*-values in MR-IVW and MR-weighted median methods were smaller than 0.05, with supportive MR-Egger. 

All estimates were reported as odds ratio (OR) per 1 standard deviation (SD) increase in vitamin B_12_ levels with their 95% confidence intervals. 5% FDR threshold was used to account for multiple testing.

#### 2.4.4. Replication

We also used disease-specific GWAS as an alternative source of MR estimates for the associations between vitamin B_12_-associated SNPs and health outcomes, when data was available. Specifically, we used the Laisk et al. meta-analysis GWAS of pernicious anaemia [25]. The summary statistics of the study were derived from a combined dataset of 2166 cases and 659,516 controls from three large population-based biobanks: the Estonian (EstBB) [26], FinnGen study, and UK Biobank cohorts. We performed IVW-MR, MR-weighted median and MR-Egger to estimate the effect of 1-SD increase in vitamin B_12_ levels on pernicious anaemia.

### 2.5. Statistical Software

All statistical analyses were implemented in R version 4.0.2 [27], PheWAS were conducted using the package *PheWAS* [28], two-sample MR was performed using *TwoSampleMR* [29]. Figures were produced using the R package *forestplot* [30].

## 3. Results

### 3.1. Vitamin B_12_ GRS-PheWAS Highlights Associations with Pernicious and Megaloblastic Anaemia

**Figure 1:**
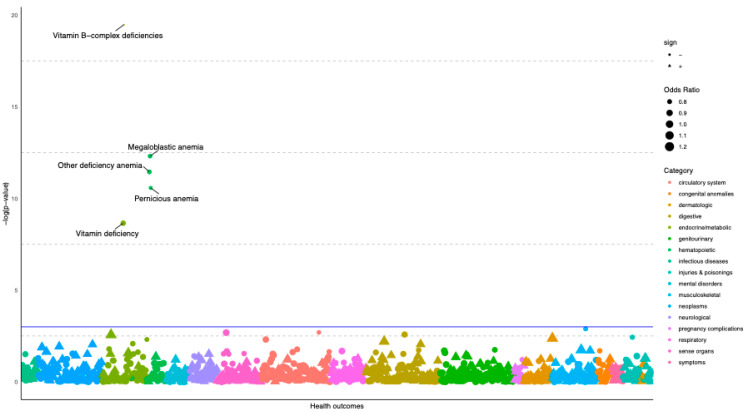
Manhattan plot depicting associations between a genetic risk score of vitamin B_12_ status and 945 health outcomes in the UK Biobank.

A total of 487,295 UK Biobank participants with a mean age of 56.9 years in 2016 were included in the analysis. PheWAS was performed across 945 clinical outcomes leading to an adjusted significance threshold of *p* < 9 × 10^−04^ (FDR 5%). Five outcomes belonging to endocrine and hematopoietic disease groups were associated with genetically predicted vitamin B_12_ status (Figure 1). These are “vitamin B-complex deficiencies”, “megaloblastic anaemia”, “other deficiency anaemia”, “pernicious anaemia” and “vitamin deficiency” (Supplementary Tables S1 and S2). 

### 3.2. Mendelian Randomisation Analyses Support Potentially Protective Effect of Genetically-Predicted Vitamin B_12_ Status on Pernicious and Megaloblastic Anaemia

**Figure 2:**
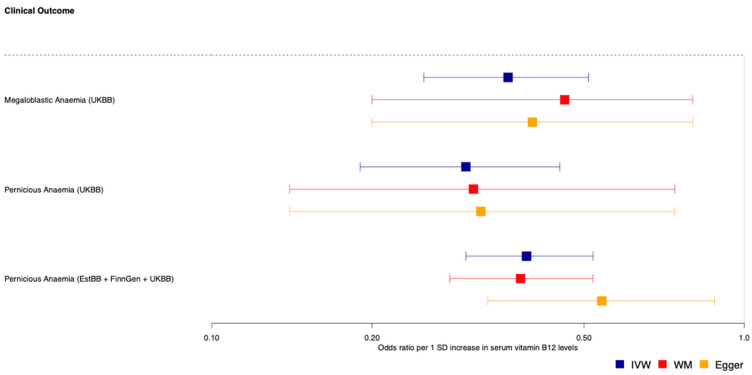
Forest plots for the Mendelian randomisation effect of genetically predicted vitamin B_12_ levels on megaloblastic and pernicious anaemia. Blue, red and yellow squares and lines depict odds ratios and 95% confidence intervals for inverse-variance weighted (IVW), weighted median (WM) and Egger MR analyses, respectively.

Results for IVW analyses and sensitivity analyses are provided in Figure 2 and Table 2. Higher genetically predicted serum vitamin B_12_ levels were found to have protective effects on vitamin B-complex deficiencies (OR = 0.22, 95% CI: 0.13–0.37), serving as a positive control in this analysis. Higher genetically predicted serum vitamin B_12_ levels were found to have a protective effect on pernicious anaemia (OR = 0.29, 95% CI: 0.19–0.45) and megaloblastic anaemia (0.35, 0.20–0.50). Sensitivity analyses were in support of IVW results for all of the studied outcomes. In fact, consistent MR estimates were obtained using the MR-weighted median method for vitamin B-complex deficiencies (0.19, 0.10–0.87), pernicious anaemia (0.31, 0.14–0.74) and megaloblastic anaemia (0.46, 0.20–0.80). MR-Egger provided less accurate estimates due to lower precision although the direction of effect for all outcomes was consistent with MR-IVW and MR-weighted median (Table 2). These results are therefore supportive of a potential causal link between higher vitamin B_12_ status and the outcomes under consideration. Pleiotropy tests showed no evidence of pleiotropy (Supplementary Table S3). The Cochran Q test showed no evidence of heterogeneity within the selected instruments (Supplementary Table S4). 

Replication analyses provided supporting evidence for the protective effects of genetically predicted serum vitamin B_12_ levels on pernicious anaemia (0.39, 0.29–0.52), with consistent effect estimates across MR methods (Table 2). The *MMA* rs2270655 SNP was excluded from the replication analysis due to inconsistent alleles in exposure and outcome datasets. There was no evidence of pleiotropy and heterogeneity within the selected instruments (Supplementary Tables S3 and S4). 

## 4. Discussion

To our knowledge, this is the first comprehensive investigation of genetically predicted vitamin B_12_ status. We provide evidence for a potentially protective effect of increased circulating vitamin B_12_ levels on vitamin B-complex deficiency, serving as a positive control for the validity of the genetic instruments used in our analyses, and on the diagnosis of pernicious anaemia and megaloblastic anaemia. 

### 4.1. MR-PheWAS Highlights Supporting Evidence of the Effect of B_12_ on Pernicious and Megaloblastic Anaemia

Pernicious anaemia is a complex autoimmune-mediated disease and the most important cause of megaloblastic anaemia. It is associated with impaired absorption of vitamin B_12_ due to the lack of intrinsic factor (IF), caused by autoimmune-mediated damage of the IF-producing gastric parietal cells (PC) lining the stomach. The aetiology of pernicious anaemia remains poorly understood although both genetic and environmental factors are thought to be important risk factors [31]. It has a significant heritable component [32] with a prevalence of 1–5 per 100,000 individuals in the UK [5] and is an important cause of morbidity. 

We highlight a risk reduction of 71% and 65% in pernicious anaemia risk and megaloblastic anaemia risk, respectively, for every SD increase in genetically predicted vitamin B_12_ status. We did not anticipate such a highly protective effect estimated from our MR-PheWAS analyses (0.39, 95% confidence interval 0.29–0.52). Whilst Laisk et al. (2021) recently highlighted a predominant role for autoimmune-related genes as risk factors for pernicious anaemia [25], results from their meta-analyses of three independent genome-wide association studies showed all the genes used in our MR-PheWAS to be significantly associated with pernicious anaemia risk, corroborating our findings as well (Supplementary Table S5). However, we believe a cautionary note is warranted in relation to diagnosis of pernicious anaemia, which is currently piecemeal. In the UK Biobank, there is a combination of either (i) individuals self-reporting as having pernicious anaemia, when in fact they may have vitamin B_12_ deficiency that is not due to autoimmune (gastritis) disease, or, (ii) individuals consulted by physicians making the diagnosis using low vitamin B_12_ levels as the sole criterion for diagnosis of pernicious anaemia. In reality, many individuals with true (autoimmune) pernicious anaemia remain undiagnosed for many years (20–30 years), sometimes indefinitely, as many general practitioners still rely solely on plasma vitamin B_12_ levels for diagnosing pernicious anaemia [33]. However, we have known for a number of years that only 10–15% of pernicious anaemia patients exhibit anaemia whilst a majority (85–90%) experience neurological and cognitive deficits without anaemia. In light of this, we purport that our instruments for genetically predicted vitamin B_12_ levels may be more powerful for diagnosing vitamin B_12_ deficiency rather than pernicious anaemia although this needs to be validated in independent, perhaps prospective, studies in the future. 

### 4.2. Strengths and Limitations

The UK Biobank study provided an excellent opportunity for us to explore the causal role of elevated vitamin B_12_ levels or deficiency across a broad spectrum of disease outcomes. We applied a hypothesis-free approach covering a broader range of disease outcomes. We did not have individual-level data on plasma vitamin B_12_ in our study cohort although the use of genetic proxies identified from other cohorts helped overcome this limitation. Our MR-PheWAS analyses were limited to traits with greater than or equal to 200 cases. Therefore, diseases with relatively low prevalence were not analysed (Supplementary Table S6). Inadequate statistical power may have also resulted in false negative results in our MR–PheWAS. For example, the previously described MR effects of genetically predicted B_12_ status on prostate and digestive system cancers were not statistically significant after correcting for multiple testing in our current analysis, although the directions of effect were consistent with previous work [16,34]. Additionally, the study was confined to individuals of European descent, thus limiting the extrapolation of our findings to other ethnic groups. We were also unable to leverage all SNPs known to be associated with vitamin B_12_ status in our GRS and MR analyses, due to the lack of their availability in our genetic dataset (*rs12272669*) or due to their pleiotropic nature. As previously mentioned, another potential shortcoming of our study is reliance on self-reported diagnosis and potentially misdiagnosis which can be prevalent among individuals with acquired vitamin B_12_ deficiency. While these limitations may affect the generalizability of our results, replication of the MR findings across several European cohorts provided additional support/validation as to the putative role of genetically determined vitamin B_12_ in the diagnosis of adverse health outcomes. 

## 5. Conclusions

Our study offers novel and previously unreported effects of genetically determined vitamin B_12_ status. We highlight a potential protective effect of higher vitamin B_12_ status on risk of pernicious anaemia and megaloblastic anaemia, albeit with the caveat that this may be due to pernicious anaemia misdiagnosis in the UK Biobank. Future research should aim to gauge the interaction between baseline vitamin B_12_ status and pernicious anaemia and megaloblastic anaemia genetic risk, to identify mechanistic pathways underlying the identified protective effect of vitamin B_12_ on megaloblastic anaemia and pernicious anaemia, and to investigate the potential use of genetically predicted vitamin B_12_ status in disease diagnosis, progression, management, and response to treatment.

## Figures and Tables

**Table 1:** Genetic instruments used for the computation of a vitamin B_12_ genetic risk score.

CHR	Gene Name	Gene Symbol	Reference SNP	Effect Allele	Other Allele	EAF	Beta	SE	*p*-Value	F-Statistic
4	Methylmalonic aciduria (cobalamin deficiency) cblA type	*MMAA*	rs2270655	G	C	0.941	0.099	0.015	5.68 × 10^−12^	46
6	Methylmalonyl-CoA Mutase	*MUT*	rs1141321	C	T	0.627	0.07	0.007	5.11 × 10^−25^	105
10	Cubulin	*CUBN*	rs1801222	G	A	0.593	0.119	0.007	7.24 × 10^−74^	329
11	Transcobalamin 1	*TCN1*	rs34324219	C	A	0.881	0.235	0.011	2.54 × 10^−109^	492
13	Citrate Lyase Beta Like	*CLYBL*	rs41281112	C	T	0.948	0.181	0.015	4.60 × 10^−34^	147
14	ATP Binding Cassette Subfamily D Member 4	*ABCD4*	rs3742801	T	C	0.294	0.053	0.007	2.28 × 10^−13^	52
19	TCII-R transcobalamin II receptor	*CD320*	rs2336573	T	C	0.031	0.313	0.019	2.89 × 10^−60^	267
22	Transcobalamin 2	*TCN2*	rs1131603	C	T	0.055	0.222	0.015	2.11 × 10^−48^	112

The statistics describing effect (beta), standard error (SE) and *p*-value are derived from the linar regression of n = 45,576 individuals conducted in Grarup et al., 2013 [12]. The effect allele is the allele associated with increased serum vitamin B_12_. F-statistics were previously calculated and extracted from Moen et al., 2018 [15]. Abbreviations: ABCD4, ATP-binding cassette subfamily D, member 4; CD320, CD320 molecule; CHR, chromosome; CLYBL, citrate lyase β-like; CUBN, cubilin; EAF, effect allele frequency; MMAA, methylmalonic aciduria (cobalamin deficiency) CblA type, MUT, methylmalonyl-CoA mutase; SE, standard error; SNP, single nucleotide polymorphism; TCN, transcobalamin.

**Table 2:** Mendelian randomisation analyses of vitamin B_12_ status and associated health outcomes.

			MR-IVW		MR-Egger		MR-Weighted Median		Outcome Cohort		
Exposure	Outcome	N_SNPs_	OR	*p*	OR	*p*	OR	*p*	Cohort	N_cases_	N_controls_
B_12_	Megaloblastic anaemia	8	0.36	1.01 × 10^−8^	0.4	4.21 × 10^−2^	0.46	5.72 × 10^−4^	UKBB	1061	384,287
B_12_	Pernicious anaemia	8	0.3	2.27 × 10^−8^	0.32	3.64 × 10^−2^	0.31	2.75 × 10^−5^	UKBB	698	384,287
B_12_	Pernicious anaemia	7	0.39	6.30 × 10^−11^	0.54	5.57 × 10^−2^	0.38	1.89 × 10^−9^	EstBB + FinnGen + UKBB	2166	659,516
B_12_	Vitamin B-complex deficiencies	8	0.22	2.46 × 10^−8^	0.29	6.84 × 10^−2^	0.19	1.97 × 10^−10^	UKBB	868	416,203
B_12_	Vitamin deficiency	8	0.45	4.58 × 10^−8^	0.45	2.93 × 10^−2^	0.45	1.55 × 10^−5^	UKBB	1734	416,203
B_12_	Other deficiency anaemia	8	0.39	6.12 × 10^−8^	0.39	3.39 × 10^−2^	0.42	9.47 × 10^−5^	UKBB	1131	384,287

## Data Availability

Summary statistics used in this analysis can be openly accessed at the IEU OpenGWAS project for the UK Biobank cohort https://gwas.mrcieu.ac.uk/ (Accessed on 5 March 2022). Pernicious anaemia meta-analysis summary statistics used for replication can be accessed at http://www.geenivaramu.ee/tools/pernicious_anemia_Laisketal2021_sumstats.gz (Accessed on 13 April 2022).

## Supplementary Materials

The following supporting information can be downloaded at: https://www.mdpi.com/article/10.3390/nu14235031/s1, Table S1: Vitamin B_12_ genetic risk score (GRS) phenome-wide association study; Table S2: SNP-based phenome-wide association study results; Table S3: Pleiotropy MR analysis results for discovery and replication cohorts; Table S4: Heterogeneity MR analysis results for discovery and replication cohorts; Table S5: Association summary statistics for genetic variants associated with pernicious anaemia from Laisk et al. 2021 [25] meta-GWAS. Table S6: Phecodes with less than 200 cases excluded from PheWAS analysis.
